# Histopathological grading of breast ductal carcinoma *In Situ*: validation of a web-based survey through intra-observer reproducibility analysis

**DOI:** 10.1186/s13000-015-0320-2

**Published:** 2015-07-10

**Authors:** Fernando Schuh, Jorge Villanova Biazús, Erika Resetkova, Camila Zanella Benfica, Alessandra de Freitas Ventura, Diego Uchoa, Márcia Graudenz, Maria Isabel Albano Edelweiss

**Affiliations:** Universidade Federal do Rio Grande do Sul (UFRGS), Porto Alegre, Brazil; Breast surgeon, Hospital de Clínicas de Porto Alegre (HCPA), Porto Alegre, Brazil; Department of Pathology, MD Anderson Cancer Center, Houston, Texas USA; Breast surgeon, Hospital São Cristóvão, São Paulo, Brazil; Department of Pathology, Hospital de Clínicas de Porto Alegre (HCPA), Porto Alegre, Brazil

**Keywords:** Classification, Ductal carcinoma *in situ*, Scoring system, Intra-observer reproducibility

## Abstract

**Background:**

Histopathological grading diagnosis of ductal carcinoma *in situ* (DCIS) of the breast may be very difficult even for experts, and it is important for therapeutic decisions. The challenge may be due to the inaccurate and/or subjective application of the diagnosis criteria. The aim of this study was to investigate the intra-observer agreement between a traditional method and a developed web-based questionnaire for scoring breast DCIS.

**Methods:**

A cross-sectional study was carried out to evaluate the diagnostic agreement of an electronic questionnaire and its point scoring system with the subjective reading of digital images for 3 different DCIS grading systems: Holland, Van Nuys and modified Black nuclear grade system. Three pathologists analyzed the same set of digitized images from 43 DCIS cases using two different web-based programs. In the first phase, they accessed a website with a newly created questionnaire and scoring system developed to allow the determination of the histological grade of the cases. After at least 6 months, the pathologists read again the same images, but without the help of the questionnaire, indicating subjectively the diagnoses. The intra-observer agreement analysis was employed to validate this innovative web-based survey.

**Results:**

Overall, diagnostic reproducibility was similar for all histologic grading classification systems, with kappa values of 0.57 ± 0.10, 0.67 ± 0.09 and 0.67 ± 0.09 for Holland, Van Nuys classification and modified Black nuclear grade system respectively. Only two 2-step diagnostic disagreements were found, one for Holland and another for Van Nuys. Both cases were superestimated by the web-based survey.

**Conclusion:**

The diagnostic agreement between the web-based questionnaire and a traditional method, both using digital images, is moderate to good for Holland, Van Nuys and modified Black nuclear grade system. The use of a scoring point system does not appear to pose a major risk of presenting large (2-step) diagnostic disagreements. These findings indicate that the use of this point scoring system in this web-based survey to grade objectively DCIS lesions is a useful diagnostic tool.

## Background

Ductal carcinoma in situ (DCIS) of the breast consists in lesions with different cytological and architectural characteristics. DCIS lesions are originated in the terminal ductolobular unit and are associated with a variable risk of invasive carcinoma development [1–4].

From a practical perspective, the precise definition of the histological grade as a predictor of biological behavior is very important, especially in regard to DCIS, because of its association with the risk of developing invasive carcinoma [5].

It is very important to establish reproducible diagnosis that can help the choice of the best treatment for each patient. Therefore, the degree of tumor differentiation is a biological variable which can be used as a prognostic factor [6]. Therapeutic decisions are made based on the histological classification, associated with other factors such as histopathological grading, size of lesion, state of margins, age of patient, mammographic correlation, and other biological markers of tumor aggressiveness assessed by molecular techniques [7–10].

Since treatment of DCIS may vary according to the potential of evolution and recurrence of the lesion, it is necessary to have clearly defined criteria to classify these lesions [11]. Considering that surgical treatment may vary from an isolated segmental resection, through a segmental resection with radiation therapy, up to a mastectomy, and given the irreversibility of therapeutic action, it is essential that the diagnosis is based on objective criteria that can be easily reproduced in daily practice [12]. Several studies have looked at the issue of diagnostic reliability and intra-observer reproducibility according to the classification studied [13–21].

Although several classification systems for DCIS have been proposed, there is only a regular level of diagnostic agreement between pathologists [14]. Many reasons may be suggested to explain this condition. Prior studies differ in how DCIS cases are presented, with variations of the origin and characteristics of the samples, core biopsy or excisional biopsies, association with invasive carcinoma, convenience or random sampling, cases with difficult grade diagnosis or representative cases. Also, there is great variation of professionals included to perform the diagnosis: some are specialists in breast pathology, while others are surgical pathologists directly involved in the diagnosis routine, not exclusively in the interpretation of breast tumors. Furthermore, instruments to gather data differ in the studies conducted so far, which contribute to the difficulty of comparing them, as well as affect the quality of reported information.

Classifications that take into account only parameters related to nuclear morphology have been proposed. These classifications have higher correlation with breast cancer biological behavior than those that take into consideration cytoarchitectural features. Therefore, they provide important clinical information with prognostic value [22].

Telepathology has been studied extensively as a mean of diagnosis and consultation in surgical pathology [23–30]. Eusebi et al. [31] have studied the telepathology diagnostic accuracy of pathologists in cases with difficult diagnosis and shown the accuracy of telepathology to be high (agreement of 75.0 %) [31]. However, before telepathology can be used confidently, thorough evaluation of its true diagnostic reproducibility is needed.

For all these reasons, this study intends to validate an electronic questionnaire available on Internet, which through a scoring point system generates the diagnosis of pathological grading of DCIS lesions in different grading systems. This study aims to assess the ability of the created questionnaire and its scoring system to reproduce the diagnosis of the pathologists in their work routine for Hollland and Van Nuys classification systems and Black modified nuclear grade.

## Methods

A cross-sectional study was carried out to evaluate the diagnostic agreement of an electronic questionnaire and its point scoring system with the subjective reading of digital images for 3 different DCIS of the breast grading systems. This project was approved by the Ethics and Research Committee at Hospital de Clínicas de Porto Alegre - number 10-247.

### Cases in the study

Slides of 43 breast DCIS cases diagnosed at Hospital de Clínicas de Porto Alegre (HCPA) and at MD Anderson Cancer Center were chosen by convenience sampling. Typical examples of DCIS were considered to select these cases. The slides selected were reviewed by two experienced pathologists (MIE and ER) without knowledge of the clinical and demographic characteristics of the patients. Cases in which there was evident invasive ductal carcinoma associated or divergence between the original anatomopathological diagnosis and the review performed at selection were excluded. The case slides were prepared from surgical specimens fixed in buffered formalin and placed in paraffin blocks, using 4 μm thick sections stained with hematoxylin–eosin.

The reviewing pathologists (MIE and ER) obtained several colored digital photomicrographs of the selected DCIS cases. The website provides images of the same field in three different magnifications (100, 200, and 400×). During analysis, the pathologist could enlarge each image provided. Each case had at least 5 images stored in JPEG format, which the observers could access freely with or without magnification.

### Participating pathologists

Three pathologists (E.R., M.G., D.U.) experts in breast pathology were invited to participate in this study. Each pathologist had specific experience with one of the study classification systems: Holland, Van Nuys and modified Black nuclear grade. They were invited to participate performing diagnoses with their preferred classification system.

### Classification systems assessed

A series of cytonuclear, cytoarchitectural characteristics and patterns of necrosis was used to compose the classification systems (Table 1).

**Table 1:** Summary of the criteria to determine the nuclear grade for the DCIS classifications studied

Holland			
	High grade	Intermediate grade	Low grade
Nuclei	Pleomorphic nuclei, anisonucleosis, irregular location, usually but not always large	Slight pleomorphism, nuclei showing some variation in size, outline and spacing	Monomorphic nuclei of uniform size, regular outline and spacing, usually small
Chromatin	Vesicular	Fine to coarse	Uniform, fine
Nucleoli	1 or more	Infrequent	No nucleoli
Mitoses	Often present	Occasionally present	Rare
**Van Nuys**			
	**High grade**	**Intermediate grade**	**Low grade**
Nuclei diameter	>2 RBC	1.5–2 RBC	1–1.5 RBC
Chromatin	Vesicular	Fine to coarse	Uniform, fine
Nucleoli	1 or more	Infrequent	No nucleoli
Comedo-necrosis	Present or absent	Present	Absent
**Black Modified Nuclear Grade**			
	**High grade**	**Intermediate grade**	**Low grade**
Nuclei	Pleomorphic nuclei, anisonucleosis, irregular location, usually but not always large	Slight pleomorphism, nuclei showing some variation in size, outline and spacing	Monomorphic nuclei of uniform size, regular outline and spacing, usually small
Nuclei diameter	Up to 3 times the diameter of normal nuclei	Up to 2 times the diameter of normal nuclei	Same as normal nuclei
Chromatin	Vesicular	Fine to coarse	Uniform, fine
Nucleoli	1 or more	Infrequent	No nucleoli
Mitoses	Often present	Occasionally present	Rare

The modified Black nuclear grade, used mainly by American pathologists in the evaluation of both invasive and intraductal breast cancer, evaluates the nuclear characteristics of breast cancers. Black and colleagues [32, 33] proposed a nuclear grading system with five grades. Contrary to common practice, grade 0 and 1 were used to designate the most poorly differentiated, or anaplastic neoplasms, whereas grade 4 reflected the well-differentiated tumors. This reversal of the numerical order remained a disturbing aspect of this nuclear grading system and contributed to a lack of wide support for its application. The nuclear-grading system of Black and colleagues has been found to be useful in predicting prognosis [34]. Fischer and coworkers devised a grading method and modified the Black nuclear grading system by reducing it from five to three grades after combining grades 0 and 1 into one group, and grades 3 and 4 into another. Furthermore, they inverted the numerical order so that grade 1 corresponds to the well differentiated carcinomas, and grade 3 reflects the most poorly differentiated tumors. In this study, the participant patologist classified cytonuclear differentiation (nuclear grade) according to criteria published [34].

Holland’s classification, used by the European Pathologists Working Group, primarily emphasizes cytonuclear differentiation and secondarily architectural differentiation (cellular polarization). This system classifies DCIS in three groups: poorly, moderately (intermediately) and well differentiated. The term ‘comedonecrosis’ is not used as a diagnostic criterion [35]. In this study, the participant assessed the criteria to compose the degree of cytonuclear differentiation (nuclear grade), as well as the cellular polarization, according to the criteria published.

The Van Nuys scale values the nuclear grade and the presence or absence of comedo-type necrosis. The presence of any high nuclear grade (with or without comedo-type necrosis) is defined as Group 3. Among the remaining non-high nuclear grade lesions, those with comedo-type necrosis are defined as Group 2, and those without comedo-type necrosis are defined as Group 1. Special types of DCIS are included in this classification [9]. The participant of this study identified both presence and absence of comedo-type necrosis and also the remaining criteria necessary to compose the nuclear grade, according to the literature published.

### Web-based model

Partially, the methodology of this study was already published in a previous paper, Schuh *et al*. [17]. We created two computer software programs which can be accessed through Internet, in website format. Every participant pathologist received a username and password to access the site. Both sites can be accessed by visitors using the login ‘patholdiagn’ and the password ‘123456’.

**Fig. 1:**
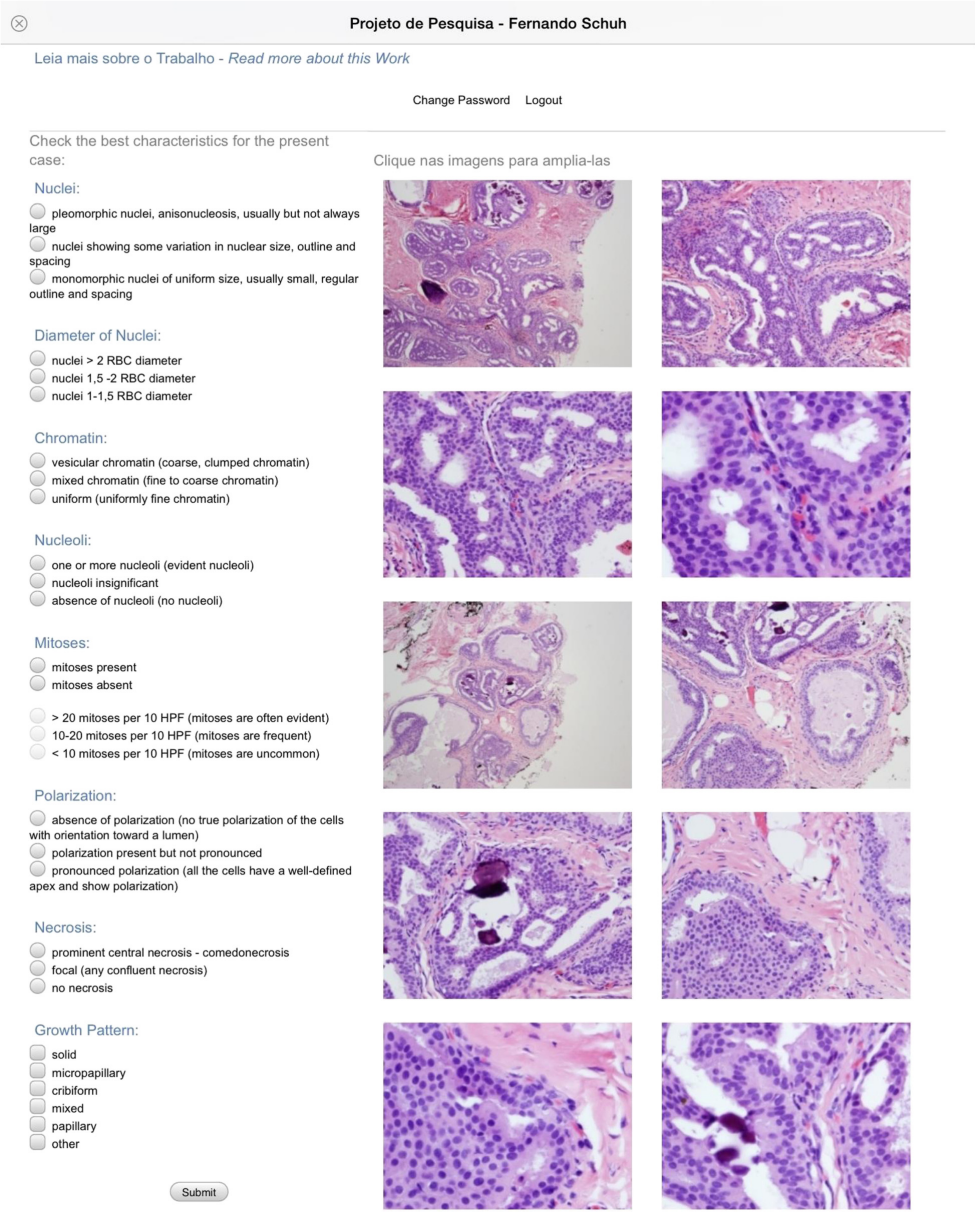
Screenshot from the website that provides the newly created questionnaire and scoring system

The three participant pathologists had accessed to the first software through the address http://www.mayer.art.br/cainsitu/site3. This program offers the digitized microscopy images of the 43 DCIS cases in study and a questionnaire containing the characteristics used to compose the three DCIS classification systems (Fig. 1). A scoring system was developed to allow the determination of the histological grade of the cases (Table 2). This electronic questionnaire and the diagnostic scoring point system compose the web-based survey that is proposed to be validated in this study. Based on the evaluation of the data obtained at the end of each case, the program itself accesses the histological grade for the three classification systems.

**Table 2:** Score for each histological finding for the generation of histological grade for the DCIS classification systems: Holland, Van Nuys and modified Black nuclear grade system

Holland	Van Nuys	Modified black nuclear grade system
**G1 if final score <6**	**G1 if final score <50**	**G1 if final score <6**
(1) monomorphic nuclei of uniform size, regular outline and spacing	(10) Nuclei 1.5 -2 RBC diameter or nuclear size in relation to normal duct: 2 fold variation in nuclear diameter	(1) monomorphic nuclei of uniform size, regular outline and spacing
	or	
(1) uniform, fine chromatin	(1) Nuclei 1-1.5 RBC diameter or nuclear size in relation to normal duct: similar, minimal enlargement	(1) Nuclei 1-1.5 RBC diameter or nuclear size in relation to normal duct: similar, minimal enlargement
(1) no nucleoli	(10) fine to coarse chromatin	(1) uniform, fine chromatin
	or	
(1) no mitoses	(1) uniform, fine chromatin	(1) no nucleoli
(1) all the cells have a well-defined apex and show polarization	(2) nucleoli insignificant	(1) no mitoses
	or	
	(1) no nucleoli	
	(10) any confluent necrosis	
	or	
	(1) no necrosis	
**G2 if final score >5 and <90**	**G2 if final score >1000 and <1030**	**G2 if final score >5 and <90 or >13 and <16 if no mitoses**
(10) nuclei showing some variation in nuclear size, outline and spacing	(10) Nuclei 1.5 -2 RBC diameter or nuclear size in relation to normal duct: 2 fold variation in nuclear diameter	(10) nuclei showing some variation in nuclear size, outline and spacing
or	or	or
(1) monomorphic nuclei of uniform size, regular outline and spacing	(1) Nuclei 1-1.5 RBC diameter or nuclear size in relation to normal duct: similar, minimal enlargement	(1) monomorphic nuclei of uniform size, regular outline and spacing
(10) fine to coarse chromatin	(10) fine to coarse chromatin	(10) nuclei 1.5 -2 RBC diameter or nuclear size in relation to normal duct: 2 fold variation in nuclear diameter
or	or	or
(1) uniform, fine chromatin	(1) uniform, fine chromatin	(1) nuclei 1-1.5 RBC diameter or nuclear size in relation to normal duct: similar, minimal enlargement
(2) nucleoli insignificant)	(2) nucleoli insignificant	(10) fine to coarse chromatin
or	or	or
(1) no nucleoli	(1) no nucleoli	(1) uniform, fine chromatin
(10) mitoses present	(1000) proeminent central necrosis - comedonecrosis	(2) nucleoli insignificant
or		or
(1) absent		(1) no nucleoli
(10) polarization present but not pronouced		(1) no mitoses
or		
(1) all the cells have a well-defined apex and show polarization		
**G3 if final score >95**	**G3 if final score >100 and <320 or >1100**	**G3 if final score >95 or >13 and <16 if mitoses are present**
(100) pleomorphic nuclei, anisonucleosis, usually but not always large	(100) nuclei >2 RBC diameter or nuclear size in relation to normal duc: 3 fold variation in nuclear diameter	(100) pleomorphic nuclei, anisonucleosis, usually but not always large
or	or	or
(10) nuclei showing some variation in nuclear size, outline and spacing	(10) nuclei 1.5 -2 RBC diameter or nuclear size in relation to normal duct: 2 fold variation in nuclear diameter	(10) nuclei showing some variation in nuclear size, outline and spacing
or	or	or
(1) monomorphic nuclei of uniform size, regular outline and spacing	(1) nuclei 1-1.5 RBC diameter or nuclear size in relation to normal duct: similar, minimal enlargement	(1) monomorphic nuclei of uniform size, regular outline and spacing
(100) coarse, clumped chromatin	(100) coarse, clumped chromatin	(100) nuclei >2 RBC diameter or nuclear size in relation to normal duc: 3 fold variation in nuclear diameter
or	or	or
(10) fine to coarse chromatin	(10) fine to coarse chromatin	(10) nuclei 1.5 -2 RBC diameter or nuclear size in relation to normal duct: 2 fold variation in nuclear diameter
or	or	or
(1) uniform, fine chromatin	(1) uniform, fine chromatin	(1) nuclei 1-1.5 RBC diameter or nuclear size in relation to normal duct: similar, minimal enlargement
(10) evident nucleoli	(10) evident nucleoli	(100) coarse, clumped chromatin
or	or	or
(2) nucleoli insignificant	(2) nucleoli insignificant	(10) fine to coarse chromatin
or	or	or
(1) no nucleoli	(1) no nucleoli	(1) uniform, fine chromatin
(10) mitoses present	(1000) proeminent central necrosis - comedonecrosis	(10) evident nucleoli
or	or	or
(1) mitoses absent	(10) any confluent necrosis	(2) nucleoli insignificant
	or	or
(100) no true polarization of the cells with orientation toward a lumen	(1) no necrosis	(1) no nucleoli
or		
(10) polarization present but not pronouced		(10) present mitoses
or		or
(1) all the cells have a well-defined apex and show polarization		(1) absent

**Fig. 2:**
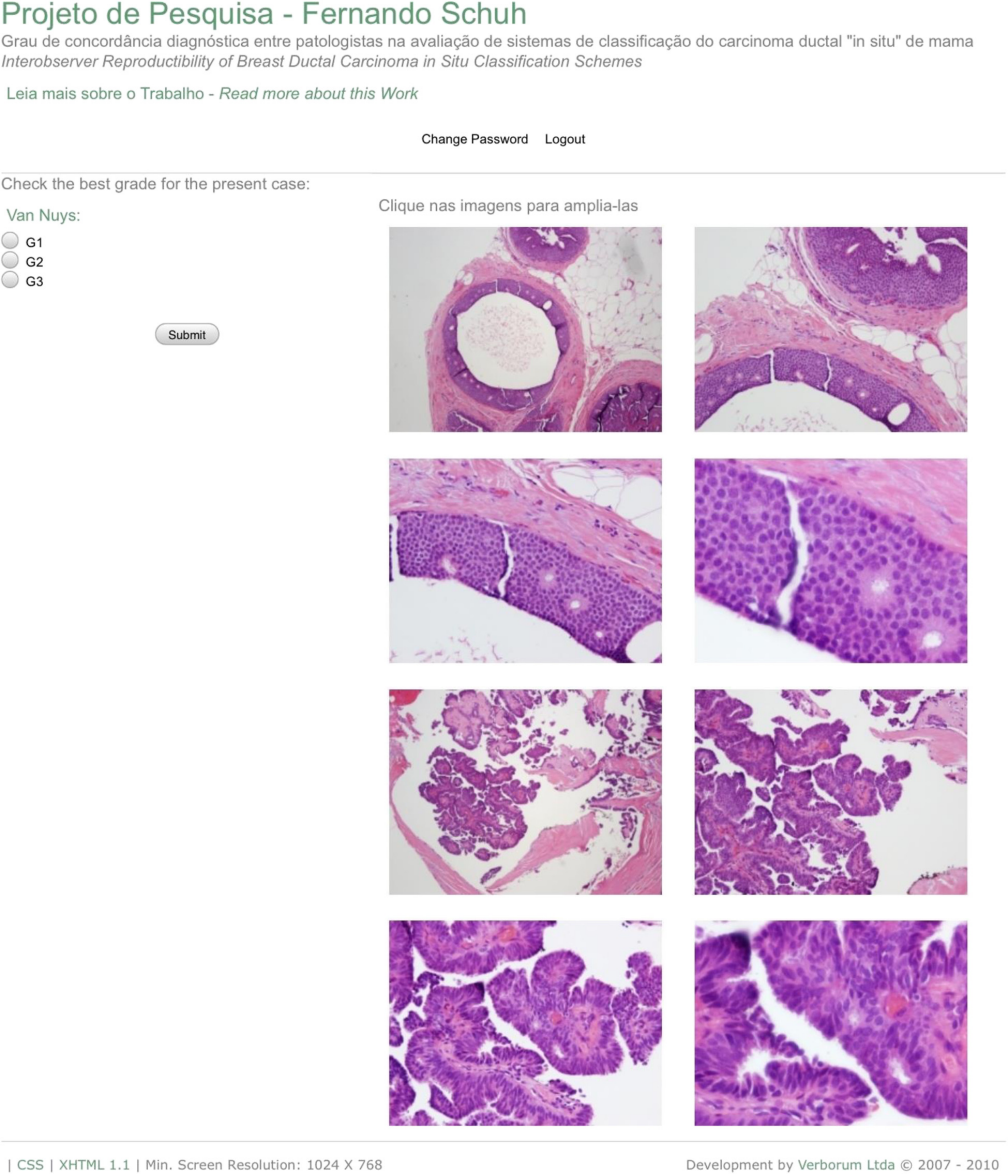
Screenshot from the website that allows subjective diagnosis, without help of the questionnaire

After at least six months, the same three pathologists were again assigned to classify the same cases of DCIS. In this phase, the pathologists accessed the second website, http://www.mayer.art.br/cainsitu/site2 (Fig. 2). In this moment, however, the final diagnosis was given by the pathologist without the aid of the questionnaire containing the criteria and scoring system for diagnosis. Each pathologist was responsible for a particular classification system.

### Statistical analysis

The Kappa statistical method was used to access the diagnosis agreement of each classification system by comparing the scoring point system and the subjective reading of the digital images on a web-based survey. Intra-observer reproducibility was calculated using Cohen’s κ statistics. Intra-observer reproducibility between the two methods (scoring point system and subjective analysis) using digital images was calculated for each pathologist [36, 37].

For each classification, the proportion of the different histologic grades found for all cases by the scoring point system and the subjective reading was also estimated.

The histological grading diagnoses were considered semi quantitative variables and were aggregated into 3 categories, with each diagnosis corresponding to a step from well differentiated to undifferentiated. The ordering of these 3 diagnostic categories was low, moderated and high grade. Any difference in diagnostic category between the two methods using digitized images was considered a diagnostic disagreement by 1 or 2 steps. Therefore, diagnoses that fell into the same category were considered concordant.

Program SPSS v.14.0 and PEPI (programs for epidemiologists) v.4.0 were used for statistical analysis of the data.

According to the sample calculation, for a 0.7 Kappa, 95 % confidence interval and 15 % margin of error, at least 43 different cases of DCIS would be needed.

## Results

Table 3 shows the proportion of cases found in each histological grade in the three classification systems studied, obtained by the diagnostic scoring system and by the subjective reading of the digital images of DCIS.

**Table 3:** Proportion of cases found in each histological grade in the three classification systems studied

Systems	Nuclear grade	Diagnostic scoring system	Subjective reading
		n (%)	n (%)
Black	Grade 1	12 (27.9)	11 (25.6)
	Grade 2	10 (23.3)	13 (30.2)
	Grade 3	21 (48.8)	19 (44.2)
Holland	Grade 1	2 (4.7)	8 (18.6)
	Grade 2	19 (44.2)	15 (34.9)
	Grade 3	22 (51.2)	20 (46.5)
Van Nuys	Group 1	17 (39.5)	17 (39.5)
	Group 2	5 (11.6)	9 (20.9)
	Group 3	21 (48.8)	17 (39.5)

The intraobserver κ values comparing the scoring point system and the subjective reading of digital images of DCIS for each of the three grading systems are shown in Table 4. A κ value of 1 reflects perfect agreement among all observers. When agreement is only by chance, the κ value is 0, and with κ <0 the observers generally disagree. Although there are no formal criteria to qualitatively describe κ values, many observers consider that κ >0.81 indicate excellent reproducibility, κ from 0.61 to 0.80 good reproducibility, κ from 0.41 to 0.60 moderate reproducibility, κ from 0.21 to 0.40 accetable reproducibility, and κ from 0 to 0.20 poor or weak reproducibility [36]. By these criteria, our results show fair to good intraobserver diagnostic reproducibility. There was no statistically significant difference between kappa values of Holland classification if compared to others (*p* = 0.317).

**Table 4:** Intraobserver reproducibility between the scoring point system and the subjective reading for the three different DCIS grading classifications studied

DCIS classification systems	Kappa values (κ ± EP)
Holland	0.57 ± 0.10
Van Nuys	0.67 ± 0.09
Black modificado	0.67 ± 0.09

Table 5 shows the degree of disagreement found in this study between the web-based survey and the subjective reading of the digital images of DCIS, for each classifcation system studied.

**Table 5:** Degree of disagreements between the web-based survey and the subjective reading in the three classification systems studied

	1-step disagreement			2-step disagreement		
	Super-estimated^a^	Sub-estimated^a^	Total	Super-estimated^a^	Sub-estimated^a^	Total
	n (%)	n (%)	n (%)	n (%)	n (%)	n (%)
Holland	8 (18.6)	2 (4.6)	10 (23.2)	1 (2.3)	0 (0.0)	1 (2.3)
Van Nuys	5 (11.6)	3 (7.0)	8 (18.6)	1 (2.3)	0 (0.0)	1 (2.3)
Black	5 (11.6)	4 (9.3)	9 (20.9)	0 (0.0)	0 (0.0)	0 (0.0)

^a^Superestimated and Subestimated by the web-based survey vs the subjective reading

Only two 2-step diagnostic disagreements were found, one for Holland and another for Van Nuys. Both cases were superestimated by the web-based survey (Grade 1 subjectively and grade 3 objectively). The case of Holland got grade 3 by the scoring point system because of nuclear grade 1 with absence of polarization. The Van Nuys case scored 3 because the nucleolus was marked as very evident.

1-step diagnostic disagreements were seen in 9 cases of modified Black grade system, 10 cases of Holland classification and 8 Van Nuys classification cases. These 1-step situations mostly have occurred by the scoring point system super estimation.

## Discussion

Two different methodological approaches have been advocated for telepathologic diagnosis. In dynamic systems, images are viewed live and in real time as the receiving viewer directly controls specimen orientation, field selection and fine focus of the microscope via robotic controls [38]. In static systems, images are captured in a digital format on an image frame grabber board and then transmitted individually as still images to the receiving viewer. The receiving viewer usually has little or no direct control over microscope functions [10, 25, 27, 28]. Although dynamic imaging is unquestionably the most powerful technological approach, the substantial lower cost favors the use of static imaging methods for review of histological slides [27], as used in this study.

When evaluating the reproducibility of diagnosis made through two or more different method diagnosis modalities, the overall percentage or proportional agreement appears to be a simple and intuitively correct measure of reproducibility. Given the limited number of diagnostic possibilities, it is important to correct for chance agreement. *Agreement* is the overall or proportional number of cases given the same diagnosis between or within observers, including that part of the agreement which may be attributable to chance. *Reproducibility,* part of the agreement that may not be explained purely by chance, is appropriately measured by the κ statistic [20]. Reproducibility can be evaluated at the level of 2 or more observers examining the same specimen (inter-observer reproducibility) or at the level of the same observer examining a specimen via 2 or more modalities or in 2 or more occasions (intra-observer reproducibility) [20].

We found that the intra-observer diagnostic reproducibility for digital images using a web-based survey was moderate to good, with κ values ranging from 0.57 ± 0,10 to 0.67 ± 0,09 for intra-observer reproducibility. Factors such as initial selection of slide fields for imaging and transmission, technical factors (digitization, transmission and display), viewer expertise and comfort when viewing and interpreting computer images seem to play a great role in determining intra-pathologist disagreements in the final diagnosis. As instrumentation improves and pathologists gain more experience in sending, receiving and interpreting digital images, the diagnostic reproducibility of digital images is likely to improve [24, 25, 27, 28]. In this study, however, what was confronted was a diagnostic scoring system with a subjective reading just using the static telepathology with the same set of digitized microscopy images of the DCIS cases.

A number of prior studies have addressed the issue of inter-observer and intra-observer reproducibility in the diagnosis of proliferative breast lesions [9, 13–17, 19, 29]. In a study examining the diagnostic accuracy of conventional examination of DCIS section slides, Douglas-Jones et al. [14] found an interobserver κ of 0,57 to 0,58 for Van Nuys classification. In that study, 19 participating pathologists reviewed all 60 cases studied [14]. Although in our study we used intraobserver concordance to compare diagnoses by the scoring system and the conventional reading of digital images, the kappa values were very similar to that study.

The diagnostic categories used in this study are semiquantitative in nature, limited to three categories: low, moderated and high grade. For quality assurance purpose, a minor discrepancy is often defined as a 1-step difference between the original and the observer diagnoses and a major discrepancy as a 2-step difference. It is a relevant aspect to be considered because differences of more than one step may be expected to result in significantly different follow-up/treatment approaches. However in this study only two 2-step diagnostic disagreements occurred, one for Holland and another for Van Nuys.

One conclusion that can be drawn from this study is that its results demonstrate for the first time that histological grading of DCIS, evaluated by three different systems, can be applied with a high degree of consistency using the available scoring point system on this innovative web-based survey. In fact, the level of observer agreement we obtained in this study was higher than that seen in prior studies of observer agreement in proliferative breast lesions [9, 13–17, 19, 29].

There are a number of potential limitations to this study. First, it could be argued that our results may not be representative of the level of agreement attainable in general pathology practice, because all pathologists participating in this study have a particular interest in breast pathology. Second, the pathologists in this study were asked to render their diagnoses following examination of selected digital images rather than following examination of whole histological sections under the microscope, as done in routine clinical practice. However, given that the goal of this study was to assess observer variability in the classification of specific lesions, we believe that the use of digital images could be viewed as a strength of the study, as it required the participants to base their diagnoses only in the microscopic features of the lesions in question, without the aid of surrounding histological clues.

## Conclusions

In summary, the intra-observer diagnostic reproducibility of DCIS with the use of digital images in a web-based survey comparing subjective analysis with the use of a point scoring system is fair to good for Holland, Van Nuys and modified Black nuclear grade system. The use of scoring point system does not appear to pose a major risk of presenting large (2-step) diagnostic disagreements. These findings indicate that the use of this point scoring system in this web-based survey to objectively grade DCIS lesions is a promising and useful diagnostic tool.
